# AAPM Task Group 103 report on peer review in clinical radiation oncology physics

**DOI:** 10.1120/jacmp.v6i4.2142

**Published:** 2005-11-22

**Authors:** Per H. Halvorsen, Indra J. Das, Martin Fraser, D. Jay Freedman, Robert E. Rice, Geoffrey S. Ibbott, E. Ishmael Parsai, T. Tydings Robin, Bruce R. Thomadsen

**Affiliations:** ^1^ Department of Radiation Oncology Middlesex Hospital 536 Saybrook Road Middletown Connecticut 06457; ^2^ Department of Radiation Oncology University of Pennsylvania 3400 Spruce Street Philadelphia Pennsylvania 19104; ^3^ CHEM Center for Radiation Oncology 48 Montvale Avenue Stoneham Massachusetts 02180; ^4^ Department of Medical Physics Hartford Hospital 80 Seymour Street Hartford Connecticut 06102; ^5^ Radiological Physics Center Department of Radiation Physics University of Texas M.D. Anderson Cancer Center 1515 Holcombe Boulevard Houston Texas 77030; ^6^ Department of Radiation Oncology Medical University of Ohio 3000 Arlington Avenue Toledo Ohio 43614; ^7^ Theragenics Corporation®, Consultant 4524 Pine Mountain Road Birmingham Alabama 35213; ^8^ Departments of Medical Physics and Human Oncology University of Wisconsin 1530 Medical Sciences Center Madison Wisconsin 53706 U.S.A.

**Keywords:** radiation oncology physics, peer review, quality assurance

## Abstract

This report provides guidelines for a peer review process between two clinical radiation oncology physicists. While the Task Group's work was primarily focused on ensuring timely and productive independent reviews for physicists in solo practice, these guidelines may also be appropriate for physicists in a group setting, particularly when dispersed over multiple separate clinic locations. To ensure that such reviews enable a collegial exchange of professional ideas and productive critique of the entire clinical physics program, the reviews should *not* be used as an employee evaluation instrument by the employer. Such use is neither intended nor supported by this Task Group. Detailed guidelines are presented on the minimum content of such reviews, as well as a recommended format for reporting the findings of a review. In consideration of the full schedules faced by most clinical physicists, the process outlined herein was designed to be completed in one working day.

PACS numbers: 87.53.Xd, 87.90.+y

## I. INTRODUCTION

A significant number of clinical physicists in the United States work as the only physicist in their department (29% of respondents in the 2002 AAPM professional survey). Task Group 11 of the Professional Information and Clinical Relations Committee recently completed its work and published its recommendations for the solo practice of radiation oncology physics (AAPM Report No. 80), with a key recommendation being an annual peer review by a qualified medical physicist.^(1)^ While peer review is particularly important for a solo physicist, we believe it is highly beneficial for all clinical radiation oncology physicists.

Peer review is gaining support as an important component in ensuring patient safety and quality of care. While most “physician extender” disciplines (such as radiation therapists) rely on continuing education criteria for renewal of registration, medical physicists are not physician extenders but function as independent professionals. This is implicitly recognized by the inclusion of the medical physics subspecialties in the American Board of Medical Specialists (ABMS), the umbrella organization for board certification of physician specialties and medical physicists. Hence, more appropriate comparison groups are our radiologist and radiation oncologist colleagues. Radiologists and radiation oncologists have been long‐time proponents of peer review through the voluntary practice accreditation programs administered by the American College of Radiology (ACR) and American College of Radiation Oncology (ACRO). The ABMS recently adopted its Maintenance of Certification program,^(2)^ the fourth component of which requires “evidence of evaluation of performance in practice.” The American Board of Radiology (ABR) recently published its Maintenance of Certification program,^(3)^ stating that one method for satisfying the fourth component is peer review. It appears, therefore, that peer review will become an increasingly common component for medical professionals in health care quality assurance.

To ensure that such reviews become a productive tool for the clinic and physicist to maintain high professional standards, the Professional Information and Clinical Relations (PICR) committee formed Task Group 103, on mechanisms for peer review in clinical radiation oncology physics. The charges for this Task Group were (1) to gather information on existing peer review processes, such as Radiological Physics Center (RPC) on‐site reviews, ACR and ACRO practice accreditation programs, and assess their relevance to a peer review between two clinical radiation oncology physicists; and (2) to formulate a framework for peer review between two clinical radiation oncology physicists, including minimum components to review and suggested criteria, as well as a suggested format of the written report summarizing the peer review.

This document is the report of Task Group 103 of the Professional Information and Clinical Relations Committee relating to the aforementioned charge, and represents the Task Group's recommendations for a voluntary peer review process between two clinical radiation oncology physicists. This report does *not* address review processes that are initiated by a physicist's employer without the explicit, and voluntarily offered, prior recommendation of the incumbent physicist.

The reviewer should, as much as practical, be independent from the reviewed physicist (e.g., no business partnership or close personal relationship), and should meet the AAPM definition of a qualified medical physicist in radiation oncology physics, which states:

For the purpose of providing clinical professional service, a Qualified Medical Physicist is an individual who is competent to practice independently one or more of the subfields of medical physics [Radiological Physics or Therapeutic Radiological Physics]. The AAPM regards board certification [ABR, ABMP or CCPM] in the appropriate medical subfield and continuing education as the appropriate qualifications for the designation of Qualified Medical Physicist. In addition to the above qualifications, a Qualified Medical Physicist shall meet and uphold the ‘Guidelines for Ethical Practice of Medical Physicists’ as published by the AAPM, and satisfy state licensure where applicable.^(4)^

It is important to recognize that the reviewed physicist, provided he/she meets the AAPM definition of a qualified medical physicist in radiation oncology physics, is an independent professional who is empowered to exercise independent professional judgment as to how to implement Task Group recommendations and codes of practice. For any given clinical physics problem, different approaches may yield similar results. Nothing herein implies a trespass upon the reviewed physicist's independent judgment in such matters, nor a diminution in responsibility for these judgments.

The AAPM believes that a properly conducted peer review can be a productive tool for the reviewed physicist to maintain high professional standards, and believes the mechanisms described in this report can help the review process. As stated above, the two physicists involved in a peer review are independent professionals, and the AAPM therefore does not endorse any specific interpretations or findings of any individual peer review.

## II. METHODS

The Task Group members were selected to represent medical physicists with experience in professional peer review programs (ACR, ACRO, and RPC), professional legal issues, solo practice and medium‐sized nonacademic clinical environments, and professional ethics. The Task Group reviewed the aforementioned peer review programs and discussed their relevance to a peer review process between two clinical radiation oncology physicists, then considered the legal and ethical aspects of such a process to define the overall scope and context of the proposed review process. Finally, practical and logistical limitations were considered in drafting a review process to fit the previously identified scope and context. This draft of a review process was then distributed to approximately 20 actively practicing clinical radiation oncology physicists (11 of whom were either in solo practice or worked as consultants for small and medium‐sized clinics) for critique and suggestions, and the document was revised to incorporate the majority of the suggestions received. The revised document was presented to the AAPM Professional Council and to the Science Council's Therapy Physics Committee for further review and suggestions. This report incorporates the Professional Council and Therapy Physics Committee's suggestions, and has been approved by the Professional Council. Finally, the document was used to perform a peer review of the Task Group chair's solo practice physics program by an independent solo practice physicist with no prior involvement in the Task Group's work, to provide a realistic test of the guidelines.

## III. RESULTS AND DISCUSSION

### A. Overview

The purpose of the peer review process, in the context of Task Group 11's recommendation,^(1)^ is to enable a collegial exchange of professional ideas and promote a productive critique of the incumbent's clinical physics program with the aim of enhancing the program while ensuring conformance with regulations, professional guidelines, and established practice patterns. In this context, the overall process would consist of the following:

1. A formal agreement with an outside, qualified medical physicist. The format of this agreement should be established in consultation with the incumbent physicist and the administrator responsible for radiation oncology and/or the medical director for radiation oncology.
2. An annual overall review, with special focus on reviews of new equipment following installation, new procedures with implementation, or a change in the medical physicist for the practice. With a completely stable practice, a less frequent schedule may be appropriate, although the time between peer reviews should not exceed three years, consistent with the ACR's and ACRO's practice accreditation frequency.
3. An on‐site visit. Some of the reviewed material, such as annual calibration reports and other documentation that does not contain patient information, could be forwarded to the reviewer in advance, reducing the time required on‐site.
4. An informal “exit interview” with the incumbent physicist. This would enable the incumbent to clarify any misunderstandings before the reviewer's report is written.
5. Written report to the reviewed physicist summarizing the findings of the review and providing suggestions for further enhancement of the physics program. The written report should be addressed to the incumbent physicist. Consistent with Task Group 11, we recommend that the physicist provide a copy of the summary to the administration and to the medical director for radiation oncology.

Recent (in accordance with the guidelines in A.2 above) successful completion of a practice accreditation review by the ACR or ACRO, or an on‐site RPC review, can be considered as fulfilling the peer review process described in this report.

In the context of this document, the term “physics group” refers collectively to the incumbent physicist, any part‐time consulting physicist(s), dosimetrist(s), in‐house radiotherapy engineer(s), and physics assistant(s).

The peer review process outlined in this report is expected to require a time commitment for the reviewer of no more than one full working day. To minimize the time required to produce a written report, we recommend that the reviewer incorporate the checklists as the “body” of the report, combined with a summary page, conclusions, and recommendations. It should be noted that the checklists are intended as tools for an expedient completion of the review process and as reminders to the reviewer of the core components to be reviewed. This does *not* imply that the incumbent physicist's performance could or should be measured by the mere existence of written procedures for each category in these checklists. A clinical physicist is an independent professional who is expected to exercise professional judgment in how best to meet the clinical physics needs of the institution and its patients, and the reviewer's assessment should be performed with this in mind; the checklists are simply tools to aid in this process.

The written report, including the summary, will be considered confidential peer review material and will be evaluated in the context of continuing professional development and quality improvement. Any use or interpretation of these reports counter to this context is inappropriate and counterproductive. The peer review process and written report are an opportunity for the physicist *and* the practice to assess how they can jointly improve the clinical physics program, and are *not* to be used in any adversarial context.

### B. Components

An effective peer review process would include the following major components: A review of the *processes* used in routine clinical physics procedures at the facility; a review of the *product* of the physics group's work, such as calibration records and patient charts; and a review of the physics *policies,* such as staffing levels and equipment maintenance.

This peer review process would involve, at a minimum, the following:

1. Independent check of treatment machines’ output calibrations (including source strength verification for high dose rate remote afterloading units). For the LINACs, the reviewer may alternatively verify that independent thermoluminescent dosimeter (TLD) output verifications have been performed during the past year, and that the results are within 5%, the RPC's criterion of acceptability in its mailed TLD program.
2. Chart audit of a *minimum* of five randomly selected recently completed treatment charts, for patients treated during the review period. The charts should be representative of the most common disease types treated in the clinic. The chart audit should include the following components^(^ ^5^ ^,^ ^15^ ^)^:
  - Verify that the dosimetry calculations were checked by a second person or second method, before the lesser of three fractions or 10% of the total dose was delivered.^(^ ^5^ ^,^ ^15^ ^,^ ^16^ ^)^
  - Verify that the chart was reviewed by the physics staff on a weekly basis.
  - Verify that the physicist reviewed the chart at the completion of treatment.^(16)^
  - Assess whether the treatment plan documents, at a minimum, localization of target and relevant normal organs, beam geometry, use of beam modifiers, beam margins around the target, choice of treatment modality and energy, choice of dose reference point and normalization, and evaluation of normal organ doses.
3. Review of the quality control and quality assurance program, using AAPM's TG‐40 as a guideline^(5)^ (as well as other Task Group reports as appropriate for specialty procedures).
4. Assessment of whether the clinical physics program is adequately documented such that another physicist could readily continue the clinic's physics services in the event of an unplanned extended absence. Clear documentation should exist for clinical dose calculations, treatment machine calibrations and routine quality control, and dosimetry equipment quality control.
5. Verification that the clinical physics program is in compliance with applicable state and federal radiation safety regulations (e.g., radioactive materials licenses, RSO designation, occupational dose limits, and review of radiation surveys for any new construction).
6. Review of the physicist's continuing professional development records (including maintenance of applicable licenses, registrations, or certifications).
7. Review of the arrangements in place for physicist coverage of extended absences by the incumbent physicist for vacations, illness, and continuing professional development.
8. Assessment of whether the existing provisions for on‐site physicist coverage are adequate for the scope of clinical services provided at the facility. Staffing level guidelines were specifically excluded from the Task Group's charge, but some recent professional society documents may be instructive: A joint European task group^(17)^ recently stressed the importance of medical physics staffing levels for quality assurance and patient safety. The European Federation of Organisations for Medical Physics issued a Policy Statement^(18)^ quantifying minimum physics staffing levels. The ACMP and AAPM commissioned an independent group to survey the medical physicist workload for commonly billed clinical procedures in the United States.^(19)^ The reviewer may wish to consult the aforementioned work, while recognizing and accounting for the different work environments in Europe and the United States.
9. Review of whether service agreements and software updates for major equipment (including, but not limited to, accelerators, imaging equipment, treatment‐planning computers, and patient management computer systems) are adequate to ensure patient safety and service continuity, and assessment of additional equipment needs consistent with the scope of clinical services being provided and/or in the process of implementation.
10. Review of the most recent peer review report, with particular focus on the report's recommendations.

### C. Checklists

**Figure 1:**
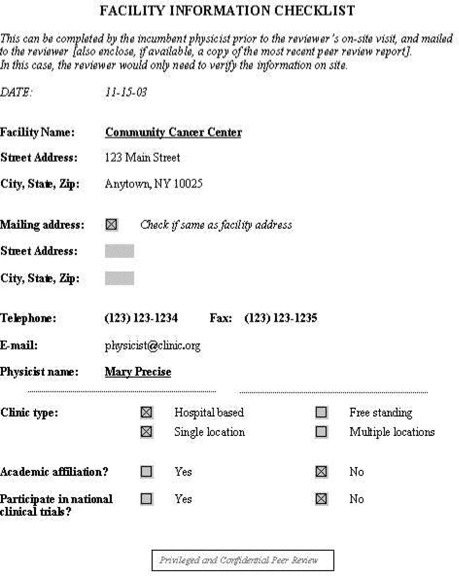
Facility information checklist. The incumbent physicist would complete this checklist and send it to the reviewer prior to the on‐site visit.

**Figure 2:**
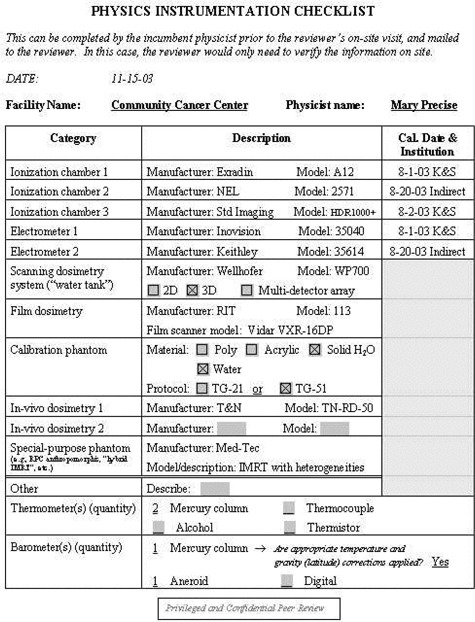
Physics instrumentation checklist. The incumbent physicist would complete this checklist and send it to the reviewer prior to the on‐site visit.

**Figure 3:**
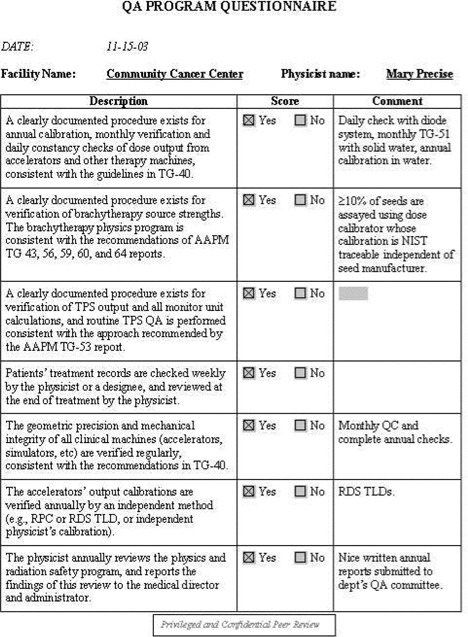
QA program questionnaire. This checklist is designed to guide the reviewer in assessing the core components of the clinical physics quality assurance program.

**Figure 4:**
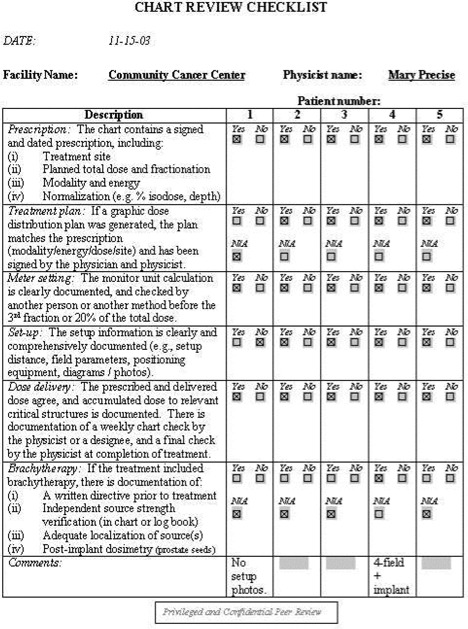
Chart review checklist. The reviewer can use this as a tool to ensure that all charts are consistently and thoroughly evaluated.

**Figure 5:**
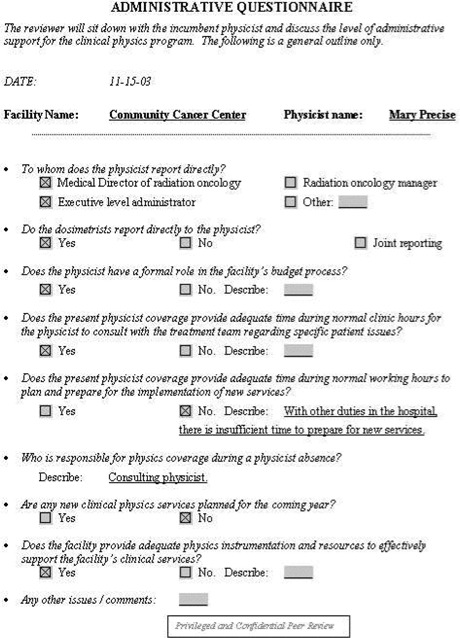
Administrative questionnaire. The reviewer can use this as a tool when evaluating the administrative structure and support for the clinical physics program.

To aid the reviewer, a set of checklists has been developed. These checklists are available as Acrobat templates for electronic completion. Sample completed checklists are shown in Figs. 1 to 5.

As stated earlier, these checklists are provided as tools for the reviewer to aid in the expedient completion of the review process and to ensure that the core components of a peer review are covered. The reviewer's *assessment* of each component outlined in the checklists should be based on how well the procedures appear to meet the specific needs of the practice and its patients. In this context, the mere presence of written procedures for each category in the checklists is not, in itself, an adequate indication of the physics program's effectiveness.

The five checklists, based on published guidelines by the AAPM and ACR^(^ ^5^ ^–^ ^16^ ^)^, are as follows:

1. Facility information: general information about the facility, such as the number of new patients treated in the past year, number of treatment machines, staffing levels, etc. See Fig. 1.
2. Equipment information: checklist of all dosimetry instrumentation. See Fig. 2.
3. QA program questionnaire. See Fig. 3.
4. Patient chart review checklist. See Fig. 4.
5. Administrative questionnaire: “interview” style, covering issues such as reporting structure, budget process, and authority delegation. See Fig. 5.

### D. Assessment of the treatment delivery chain

**Figure 6:**
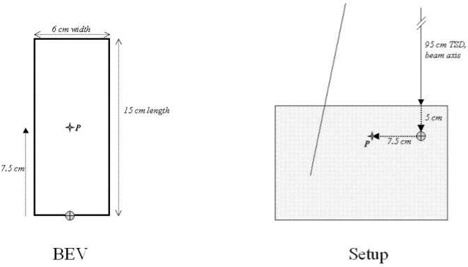
Benchmark case. This case can be used to assess the overall treatment delivery chain, including treatment planning and setup on the treatment machine.

In addition to the minimum components outlined in section B above, a test of the dose calculation and treatment delivery chain is recommended. This may help clarify any discrepancy in treatment‐planning system (TPS) beam data or in the treatment planner's use of the system. We suggest that the benchmark case described below, and illustrated in Fig. 6, be calculated by the routine treatment planner (dosimetrist or physicist), then set up by the incumbent physicist and measured by the reviewer. Agreement within ±3% would be expected for measurement with a calibrated ionization chamber, and agreement within ±5% would be expected for a properly calibrated in vivo dosimeter (diode, MOSFET, TLD).

If this test has been performed during a previous peer review, or if the RPC or Quality Assurance Review Center (QARC) benchmark cases have previously been completed, there would be no benefit in performing this test unless significant changes in TPS or accelerator equipment have occurred since the test was previously performed.

### D. 1 *Benchmark case*

Collimator setting 6.0 cm width symmetric, 15.0 cm length asymmetric half‐field. Target‐to‐surface distance 95.0 cm along the beam's central axis, incident on a flat phantom of minimum dimensions $25.0\times 25.0\times 15.0\;{\text{cm}}^{3}$. Measurement point at a depth of 5.0 cm in the phantom, at a location in the center of the effective field (7.5 cm from the beam central axis along the field's length). See Fig. 6. Calculate the monitor setting to deliver 200.0 cGy to the measurement point; a separate calculation and measurement of each photon beam is recommended.

### E. Written report

The reviewer should provide a written report to the reviewed physicist within one month of the on‐site visit. The completed checklists may be used to form a significant portion of the written report. The report should be written in the context of constructive collegial critique on how the physics program could be further enhanced. Thus, in addition to the completed checklists the report should contain the following:

1. A cover page showing the date(s) the peer review was conducted, the date of the written report, and contact information for the reviewer so that the reviewed physicist can easily follow up to clarify any suggestions in the report.
2. A two‐part summary:
  - **Major recommendations.** Major recommendations should be items that, in the reviewer's opinion, do not presently meet applicable regulations or generally accepted guidelines (such as Refs. 5 to 10 and 12 to 16), or scenarios that could result in delivered dose to the patient being in error by >5%.
  - **Minor recommendations** would be items that have no impact on regulatory compliance or generally accepted guidelines, but could enhance the physics program's productivity or level of organization/documentation.

**Figure 7:**
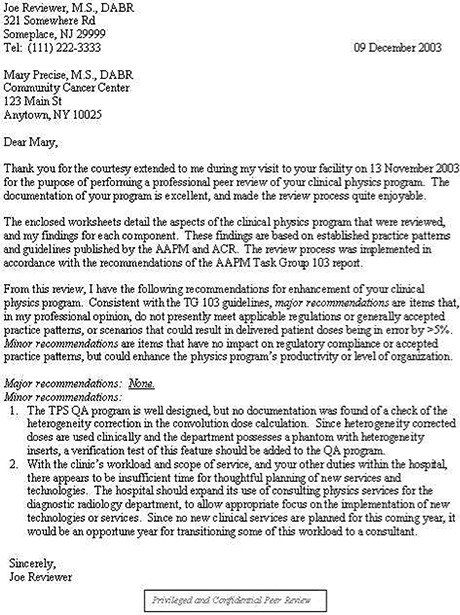
Sample summary letter. Illustration of a reviewer's concise summary of findings and recommendations for improvement.

The cover page and summary may be combined into one document, as shown in the example in Fig. 7.

We recommend that the written report be labeled with the words “Privileged and Confidential Peer Review” in the header or footer to clearly identify the confidential context of the document.

Consistent with AAPM Report #80, the Task Group strongly recommends that the reviewed physicist provide a copy of the summary document to the administrator responsible for radiation oncology and to the medical director of radiation oncology. This could be presented as a valuable component of the physicist's annual report on the clinical physics program.

### F. “Real‐life” test of peer review process

The process described herein was used to conduct a peer review of the Task Group chair's physics program in December 2004. The reviewer was an experienced clinical radiation oncology physicist in solo practice in the same state, who had not participated in the Task Group's work. The reviewed site has a single modern multi‐energy LINAC and maintains active intensity‐modulated radiotherapy and prostate seed implant programs. The reviewer was provided with the draft Task Group report and the five checklists. The reviewer and incumbent physicist exchanged information by e‐mail and telephone prior to the scheduled site visit in order to minimize the reviewer's time on site. The reviewer chose to use recent independent TLD results as verification of appropriate output calibration, thus saving time on site. The total time spent on site was 7 hours, and the written report was forwarded to the incumbent physicist one week after the on‐site visit. The reviewer estimated that an additional 2 hours were spent after the site visit to compile the results and finish the report. No significant logistical or process problems were identified with the review guidelines.

## IV. CONCLUSION

Effective peer review is an important tool for improving the clinical physics program, enhancing patient safety, and aiding the clinical physicist's professional development. The Task Group has designed a peer review mechanism it believes can be accomplished in a reasonable amount of time and enables a collegial exchange of professional ideas and productive critique of the entire clinical physics program. While the Task Group's main focus was on peer review for physicists in solo practice, we believe this document could also be the basis for a peer review process in larger groups, particularly when dispersed among multiple physical locations.

To ensure that the peer review is conducted in a productive environment, the reviewer must remember that for any given clinical physics problem, different approaches may yield similar results and that the review should not trespass upon the reviewed physicist's independent judgment in such matters, provided the results meet generally accepted guidelines.^(^ ^5^ ^–^ ^10^ ^,^ ^12^ ^–^ ^16^ ^)^ Similarly, the reviewed physicist's employer must respect the confidential nature of the peer review and the context of the reviewer's recommendations.

## ACKNOWLEDGMENTS

The Task Group would like to thank the Professional Council, and the Therapy Physics Committee of the Science Council, of the American Association of Physicists in Medicine for their constructive review and substantive suggestions in the preparation of this report.

## Supporting information

Supplementary Material Files
