# Supplementary material for: AAPM Task Group 103 report on peer review in clinical radiation oncology physics
Source: J Appl Clin Med Phys. 2005 Nov 22;6(4):50–64. doi: 10.1120/jacmp.v6i4.2142 (PMC5723459; doi:10.1120/jacmp.v6i4.2142)
Supplement: Supplementary file 1 — Supplementary Material Files [file ACM2-6-050-s001.dot]

# AAPM TG103 Report

## QA PROGRAM QUESTIONNAIRE

*DATE:*

#### Facility Name:       Physicist name:

| Description | **Score** | **Comment** |
| --- | --- | --- |
| A clearly documented procedure exists for annual calibration, monthly verification and daily constancy checks of dose output from accelerators and other therapy machines, consistent with the guidelines in TG-40 (ref. 2). | Yes  No |  |
| A clearly documented procedure exists for verification of brachytherapy source strengths. The brachytherapy physics program is consistent with the recommendations of AAPM TG 56, 59, 60, and 64 reports (ref. 3-6). | Yes  No |  |
| A clearly documented procedure exists for verification of TPS output and all monitor unit calculations, and routine TPS QA is performed consistent with the approach recommended by TG-53 (ref. 9). | Yes  No |  |
| Patients’ treatment records are checked weekly by the physicist or a designee, and reviewed at the end of treatment by the physicist. | Yes  No |  |
| The geometric precision and mechanical integrity of all clinical machines (accelerators, simulators, etc) are verified regularly, consistent with the recommendations in TG-40. | Yes  No |  |
| The accelerators’ output calibrations are verified annually by an independent method (e.g., RPC or RDS TLD, or independent physicist’s calibration). | Yes  No |  |
| The physicist annually reviews the physics and radiation safety program, and reports the findings of this review to the medical director and administrator. | Yes  No |  |

*Privileged and Confidential Peer Review*
